# SGLT2 inhibitors as metabolic modulators: beyond glycemic control in type 2 diabetes

**DOI:** 10.3389/fendo.2025.1601633

**Published:** 2025-06-24

**Authors:** Qian Wu, Jian Zhang, Fuli Zhang, Dongnan Li

**Affiliations:** ^1^ Graduate School, Heilongjiang University of Chinese Medicine, Harbin, Heilongjiang, China; ^2^ Heilongjiang University of Chinese Medicine, School of Basic Medical Sciences, Harbin, Heilongjiang, China

**Keywords:** SGLT2is, metabolic regulation, type 2 diabetes, cardiovascular protection, combination therapy

## Abstract

Type 2 diabetes mellitus (T2DM) is a complex metabolic disorder, and its management has evolved from mere glycemic control to multitarget metabolic regulation. Sodium–glucose cotransporter 2 inhibitors (SGLT2is) have demonstrated extensive pleiotropic effects in treating T2DM, and its complications through unique mechanisms. SGLT2is promote urinary glucose excretion, leading to a negative energy balance that triggers lipid metabolic reprogramming and fuel switching in the body. This process significantly reduces visceral fat deposition and improves insulin resistance and the inflammatory status. Additionally, SGLT2is provide a metabolic foundation for cardiovascular, hepatic, and renal protection through multiple pathways, including remodeling cardiac structure, enhancing myocardial metabolism, reducing uric acid levels, and alleviating renal hypoxia. With respect to combination therapy, the pairing of SGLT2is with other hypoglycemic agents and cardiovascular protective drugs has synergistic effects; however, potential adverse reactions should also be considered. Future research should investigate the precise application and long-term safety of SGLT2is as well as develop individualized treatment strategies on the basis of patients’ metabolic phenotypes, complications, and drug tolerability to maximize clinical benefits for patients. This review systematically explores the significant roles of SGLT2is in metabolic regulation, cardiovascular protection, and combination therapy, with the aim of providing a comprehensive foundation for optimizing individualized treatment strategies in T2DM management.

## 1 Introduction

Diabetes has emerged as a significant public health challenge globally in the 21st century, with its widespread prevalence not only imposing a heavy disease burden but also exerting substantial socioeconomic pressure (1). Epidemiological forecasts predict that the number of diabetes patients worldwide will rise to 578 million by 2030 (2). Among the types of diabetes, type 2 diabetes mellitus (T2DM) is the most prevalent, and its pathogenesis is complex, involving dysregulation of glucose metabolism in the body due to the interaction of various factors, such as the social environment and genetics (3). The complications of T2DM combined with cardiovascular or kidney disease increase the risk of hospitalization and mortality. The previous treatment plan for T2DM focused on restoring pancreatic beta-cell function and supplementing insulin injections, as one of the important mechanisms of T2DM is insulin resistance. Sodium–glucose cotransporter 2 inhibitors (SGLT2is) achieve glucose-lowering effects through a unique renal mechanism of action. They selectively inhibit the reabsorption of glucose in the renal tubules, promoting urinary glucose excretion independent of pancreatic β-cell function. Additionally, SGLT2is can improve the uptake and utilization of glucose in peripheral tissues and increase insulin sensitivity (4, 5). Several landmark large-scale clinical trials, including EMPA-REG OUTCOME, CANVAS, and DECLARE-TIMI, have demonstrated that SGLT2is can significantly reduce the risk of cardiovascular death and improve cardiorenal outcomes in the T2DM population (6–8).

The specific molecular mechanisms by which SGLT2is exert these multiple metabolic benefits remain an important area of current research. A growing body of evidence suggests that SGLT2is have significant pleiotropic effects on weight management, adipose tissue remodeling, cardiovascular protection, and renal protection. The focus of this review is the metabolic benefits of long-term SGLT2i treatment, its correlation with reductions in fat mass, changes in adipokines and lipoprotein profiles, and its cardiovascular benefits and hepatic and renal protective effects in relation to visceral fat and inflammatory factors. Finally, we propose that personalized treatment strategies and future research directions should be emphasized.

## 2 Metabolic regulatory effects of SGLT2is

### 2.1 Weight management and adipose tissue remodeling

SGLT2is inhibit the SGLT2 receptor in the proximal tubule of the kidney, promoting urinary glucose excretion (approximately 60–90 grams of glucose per day), resulting in an energy loss of approximately 200–300 kcal daily, which mimics a low-calorie state in the body. This negative energy balance can sometimes trigger compensatory increases in food intake, thereby reducing the anticipated weight loss effect (9). However, related studies indicate that this compensatory response does not significantly affect the efficacy of SGLT2is (10–12). The weight loss effect of SGLT2is is typically assessed through changes in BMI. An elevated BMI is typically associated with obesity, and prolonged obesity is often closely linked to high-sugar and high-fat diets. Poor dietary habits serve as a potential driving force for the development and progression of metabolic diseases. A series of pathological changes induced by obesity in the body, ranging from microvascular and macrovascular lesions to organ pathological alterations, are potential factors that exacerbate disease progression. For example, pathophysiological changes such as chronic low-grade inflammation, abnormal lipid metabolism, and insulin resistance associated with obesity collectively form the important pathogenic basis for heart failure with preserved ejection fraction (HFpEF) (13). In clinical practice, BMI is frequently used as a significant indicator for disease risk assessment. For example, studies by Said et al. showed that higher BMI can serve as an independent predictor of new-onset heart failure (HF) in patients with T2DM (14). However, BMI cannot reflect changes in body fat distribution or variations in the weight of different parts of the body, such as skeletal muscle and muscle mass. Analyses of the DELIVER, EMPEROR-Reserved, and CANDLE trials indicated that although patients with higher BMIs experience more significant symptom improvement (15), the therapeutic effects of SGLT2is in reducing the risk of cardiovascular death and other aspects are independent of baseline BMI (15–17).

The weight loss induced by SGLT2is is associated primarily with a reduction in water content and a decrease in fat mass (18). Initially, due to increased urine output, the reduction in water content is more pronounced; however, the long-term weight loss effect is related to a reduction in fat mass. In a 12-week randomized controlled trial, dapagliflozin was associated with weight loss related to a decrease in lean body mass and body water content, with no significant change in fat mass observed (19). However, in longer-term treatments (such as 24 weeks and 102 weeks), dapagliflozin not only reduced body water content but also significantly decreased fat mass (with reductions of 1.48 kg and 2.80 kg, respectively), and these effects were correlated with the duration of treatment (20, 21). The reduction in fat mass is partly attributed to the decrease in visceral adipose tissue and subcutaneous adipose tissue (22–24). SGLT2is can reduce liver fat (25, 26), the thickness of epicardial adipose tissue (EAT) (27, 28), and perirenal fat (29), among other types of tissue.

SGLT2is can alter adipose tissue structure by converting white adipose tissue into brown adipose tissue (BAT) or beige adipose tissue through browning, thereby increasing energy expenditure to adapt to the negative energy balance induced by SGLT2is. Studies have shown that SGLT2is can reduce lipid content in perirenal WAT, inguinal WAT, and epididymal WAT in mice and upregulate the expression of uncoupling protein 1 (UCP1) (30–32), thermogenesis-related genes (such as Prdm16 and Irisin) (32), and the mRNA expression of beige adipose-selective genes (such as Cd137 and Tmem26) (31). Uncoupling protein 1 (UCP1) is a crucial thermogenic regulator that can uncouple substrate oxidation from ATP synthesis, thereby generating heat. The increased expression of UCP1 reflects an increase in BAT quantity and active mitochondrial function (33). Although beige adipose tissue does not contribute to overall energy expenditure as much as BAT does, it has additional metabolic benefits in terms of glucose and lipid clearance as well as anti-inflammatory effects (34).

### 2.2 Lipid metabolism reprogramming and liver protection

**Figure 1:**
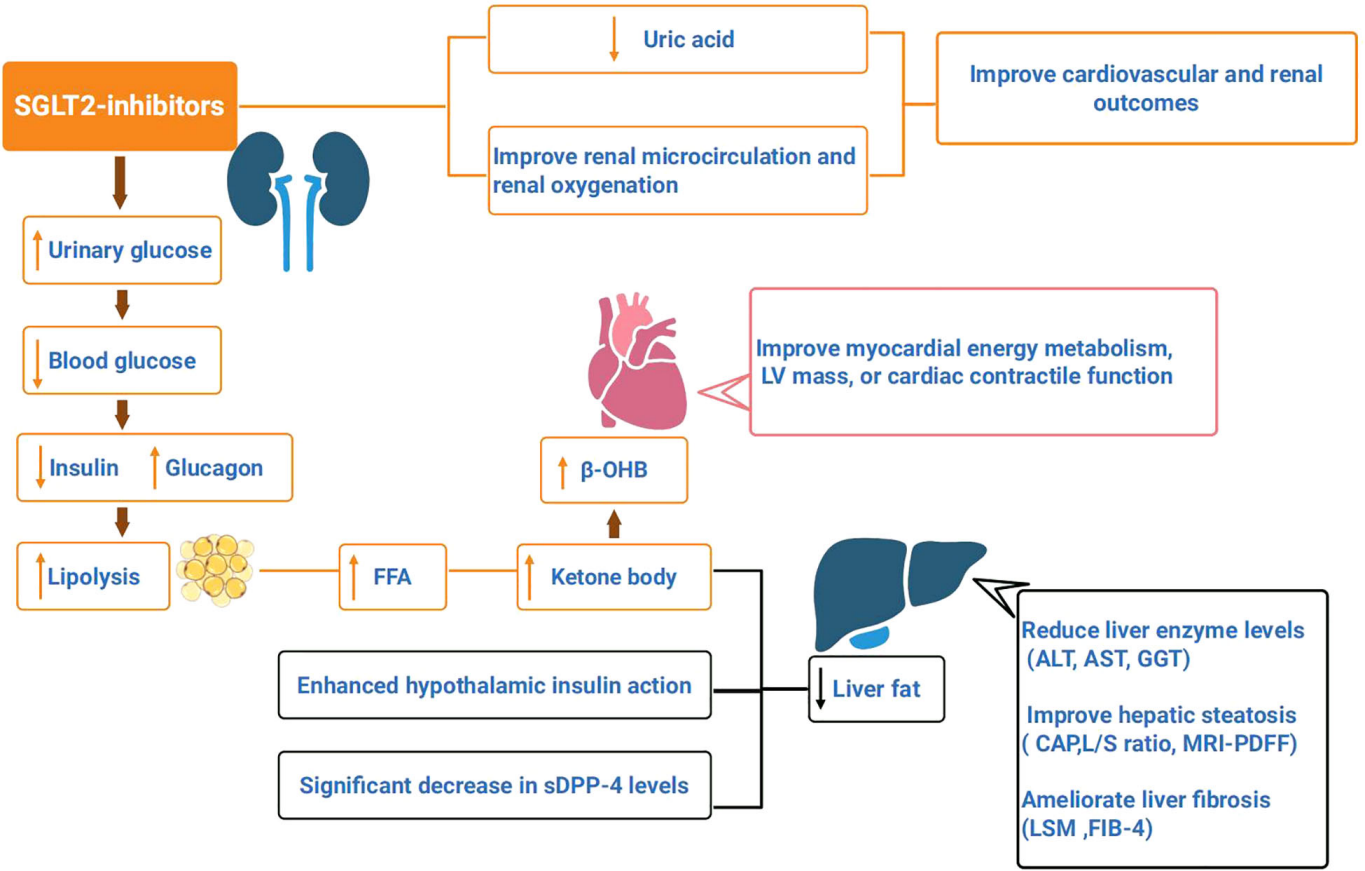
SGLT2is promote urinary glucose excretion, reduce blood glucose concentration. Reduced insulin secretion and increased glucagon can promote fat breakdown, which produces a large amount of free fatty acids (FFA), that are oxidized by liver fatty acids to form ketone bodies, and reduce liver fat. Enhanced insulin action in the hypothalamus, along with a significant decrease in serum soluble dipeptidyl peptidase-4 (sDPP-4) levels, can lead to a reduction in liver fat. SGLT2is have been shown to reduce liver enzyme levels (ALT, AST, GGT) in patients with nonalcoholic fatty liver disease(NAFLD), improve hepatic steatosis (controlled attenuation parameter (CAP), L/S ratio, MRI-PDFF), and ameliorate liver fibrosis (liver stiffness measurement (LSM), FIB-4 index). SGLT2is lead to an elevated circulating level of β-hydroxybutyrate (β-OHB), thereby improving myocardial energy metabolism, LV mass, or cardiac contractile function. SGLT2is can reduce uric acid levels, improve renal microcirculation and oxygenation, and improve cardiovascular and renal outcomes in T2DM.

SGLT2is decrease insulin secretion, and increase glucagon secretion, thereby improving β-cell function and tissue sensitivity to insulin. Substrate utilization for body metabolism shifts from carbohydrates to lipids (35). The reduction in insulin and increase in glucagon reflect the decreased inhibition of lipolysis and increased activation of hormone-sensitive lipase (HSL), which promotes lipolysis. The activation of the sympathetic nervous system stimulates β-adrenergic receptors, thereby promoting lipolysis. Although the substantial loss of glucose increases hepatic gluconeogenesis, leading to an increase in endogenous glucose production, the ultimate reduction in blood glucose concentration indicates a general shift in the body toward lipid metabolism. Fat breakdown releases a large amount of free fatty acids (FFAs), which are converted into ketone bodies through hepatic fatty acid oxidation. This process reduces lipid accumulation in the liver (**Figure 1**).

The improvement of insulin resistance also requires long-term treatment with SGLT2is. Studies have shown that 4 weeks of empagliflozin treatment has no significant effect on skeletal muscle free fatty acid or glucose uptake (18). After 8 weeks of dapagliflozin treatment, no significant improvement in insulin sensitivity was observed in tissues such as the liver, skeletal muscle, or myocardium (25). However, 12 weeks of empagliflozin treatment increased hepatic insulin sensitivity (26). A recent meta-analysis by Li et al. reported that SGLT2is can improve insulin resistance in patients with T2DM complicated by nonalcoholic fatty liver disease (NAFLD), significantly reducing HOMA-IR levels (MD [95% CI]; -0.66 [-0.99, -0.32], p = 0.0001). The duration of treatment in the 11 included studies was more than 12 weeks, with the longest duration reaching 72 weeks (36). Additionally, studies have shown that combination therapy with dapagliflozin and exenatide (EXE) can reduce hepatocellular lipids (HCLs) (-4.4%, P < 0.05), and changes in HCLs are associated with a reduction in visceral adipose tissue, independent of glycemic control (37).

SGLT2is can improve the levels of adipokines and inflammatory factors, but the benefits of SGLT2is remain uncertain. A study by Dihoum et al. revealed that dapagliflozin significantly reduced CRP after 12 months of treatment (mean difference -1.96; 95% CI -3.68 to -0.24, p = 0.026), but other inflammatory factors (tumor necrosis factor α (TNF-α), interleukin-1β (IL-1β), IL-6, and interleukin 10 (IL-10)) were not significantly improved (38). A meta-analysis by Buttice et al. demonstrated that SGLT2is significantly increased adiponectin and reduced IL-6 and tumor necrosis factor receptor-1 (TNFR1), but there was no significant change in CRP levels (39). The anti-inflammatory mechanism of SGLT2is is controversial, as their anti-inflammatory effects are not prominent in some studies and involve complex physiological mechanisms. However, the reduction in inflammatory factors may be associated with a decrease in fat mass. For example, increased hepatic fatty acid oxidation increases circulating ketone body levels, and serum β-hydroxybutyrate can inhibit NLRP3 inflammasome activation, thereby reducing IL-1B levels (40). Additionally, fat and muscle are significant sources of IL-6 (41). Although studies have shown that a reduction in IL-6 is associated with higher baseline HbA1c levels (42), this may be because IL-6 can lead to increased hepatic glucose output, subsequently increasing blood glucose levels (43).

SGLT2is can improve the lipoprotein profile, with beneficial changes in the human body being an increase in high-density lipoprotein cholesterol (HDL-C) and a decrease in triglyceride (TG) levels (36, 44). These changes are often common, but research findings can sometimes be contradictory, particularly regarding changes in low-density lipoprotein cholesterol (LDL-C). Bechmann et al.’s meta-analysis of 60 RCTs, which were all the RCTs of SGLT2is published at that time, reported an increase in total cholesterol (TC) and LDL-C (44). Elevated LDL-C levels are harmful to the body and promote the development of atherosclerotic cardiovascular disease (ASCVD) (45). Rizos et al. reported that SGLT2is do not appear to improve arterial stiffness, with no favorable changes observed in either pulse wave velocity (PWV) or the augmentation index (AIx) (46). However, numerous studies have demonstrated that SGLT2is can reduce ASCVD events in patients with T2DM (47–49). Additionally, the meta-analysis by Li et al. did not reveal significant changes in TC or LDL-C, with the majority of patients included in this study also suffering from NAFLD and T2DM (36). Current evidence suggests that SGLT2is offer substantial cardiovascular benefits for patients with NAFLD.

Furthermore, SGLT2is reduce hepatic fat, thereby lowering the risk in patients with NAFLD. Enhanced insulin action in the hypothalamus (50), along with a significant decrease in serum soluble dipeptidyl peptidase-4 (sDPP-4) levels (51), can lead to a reduction in liver fat. sDPP-4 is secreted by hepatocytes and promotes hepatic fat synthesis by degrading glucagon-like peptide-1 (GLP-1) while also inducing adipose tissue inflammation and insulin resistance. In summary, SGLT2is promote lipolysis while simultaneously inhibiting hepatic fat synthesis. Moreover, SGLT2is have been shown to reduce liver enzyme levels (ALT, AST, GGT) in patients with NAFLD (36, 52, 53), improve hepatic steatosis (controlled attenuation parameter (CAP), L/S ratio, MRI-PDFF), and ameliorate liver fibrosis (liver stiffness measurement (LSM), FIB-4 index) (36, 53–56). It has been proposed that SGLT2is could serve as the first-line treatment for patients with T2DM complicated by NAFLD (26, 57).

### 2.3 Energy metabolism and fuel conversion

According to the “thrifty substrate” hypothesis, SGLT2is lead to an elevated circulating level of β-hydroxybutyrate (β-OHB), which then competes with acetyl-CoA from FFA oxidation and glucose-derived pyruvate for entry into the tricarboxylic acid (TCA) cycle. As an energy substrate, β-OHB consumes less oxygen, thereby increasing myocardial efficiency while reducing reactive oxygen species generated by excessive FFA oxidation rates, lowering oxidative stress, and improving mitochondrial function (58). However, some studies have raised doubts about the “thrifty substrate” hypothesis. Although elevated circulating levels of β-OHB increase the likelihood of myocardial substrate utilization, they do not significantly improve myocardial energy metabolism, LV mass, or cardiac contractile function. For example, a study by Gaborit et al. showed that after 12 weeks of treatment with empagliflozin, although the liver fat content (LFC) was significantly reduced by 27%, there was no significant effect on myocardial or epicardial fat, and myocardial energetics (PCr/ATP) also did not significantly change (59). A study by Pietschner et al. demonstrated that after 12 weeks of empagliflozin treatment in patients with stable chronic heart failure (CHF), although there was an overall reduction in blood pressure and improvement in vascular function, the increase in β-OHB levels partially offset these benefits (60). Another study showed that empagliflozin treatment for one month did not alter ketone body concentrations in patients hospitalized with acute HF, further confirming that SGLT2is do not increase the risk of diabetic ketoacidosis, as the initial phase of acute HF is already accompanied by a significant increase in circulating total ketone body (TKB) concentrations (61).

Overall, elevated ketone body levels may not significantly improve cardiac function in the early stages, but in most cases, they do not impair cardiac function or increase the risk of adverse cardiac events, demonstrating good safety. Cardiac energy metabolism is highly flexible and can switch between different energy sources on the basis of substrate availability. For example, one of the characteristics of heart failure with a reduced ejection fraction (HFrEF) is an increased reliance on ketone bodies in the context of reduced fatty acid and glucose oxidation (62). Oldgren et al. reported that in patients with type 2 diabetes without heart failure, although dapagliflozin treatment for 6 weeks did not increase cardiac fatty acid uptake or improve myocardial efficiency, it significantly reduced LV work (-0.095 [-0.145, -0.043] J/g/min) and LV oxygen consumption (-0.30 [-0.49, -0.12] J/g/min) (63). Although existing evidence suggests limitations to the “thrifty substrate” hypothesis, the potential role of ketone bodies in cardiovascular benefits cannot be denied.

### 2.4 Uric acid metabolism and oxidative stress

One of the mechanisms by which SGLT2is protect the kidneys is through reducing plasma uric acid (UA) levels, but this effect is not achieved by decreasing UA production but rather by increasing UA excretion (64). This process primarily involves urate transporter 1 (URAT1) and glucose transporter 9 (GLUT9). However, the effects of SGLT2is on URAT1 and GLUT9 remain controversial. For example, SGLT2is do not increase urate excretion in URAT1-deficient mice, and GLUT9 appears to be nonessential for the urate excretion effect of canagliflozin (65). When empagliflozin is combined with the URAT1 inhibitor benzbromarone, the effect is inferior to that of benzbromarone alone (66). Although the significant role of URAT1 in renal uric acid reabsorption cannot be overlooked, the uricosuric effect of SGLT2is may rely more on GLUT9 isoform 2. For example, studies have shown that luseogliflozin does not directly affect the activity of proteins such as URAT1, GLUT9 isoform 1, and OAT4, but an increase in the luminal glucose concentration stimulates GLUT9 isoform 2 and inhibits its uric acid reabsorption (67).

However, multiple studies have also indicated that the effects of SGLT2is on reducing UA levels are associated with baseline HbA1c levels: in patients with lower baseline HbA1c and higher baseline UA levels, the reduction in UA levels is more significant (68–71). This finding appears to contradict the role of GLUT9 in reducing uric acid reabsorption through a glucose-dependent mechanism. In summary, the mechanism by which SGLT2is promote uric acid excretion may not depend entirely on transporters expressed in renal tubular epithelial cells (such as URAT1 and GLUT9) but rather involves more complex physiological processes.

SGLT2is exert significant cardiovascular and renoprotective effects by reducing UA levels. Studies have shown that SGLT2is, when improving cardiovascular outcomes in patients with T2DM, are often accompanied by a significant decrease in UA levels, including heart failure (72–74), acute myocardial infarction (AMI) (75), and gout outcomes in atherosclerotic cardiovascular disease patients (76). Notably, the ability of SGLT2is to reduce UA levels and the risk of cardiovascular events is independent of the patient’s heart failure status (77). A recent meta-analysis revealed that SGLT2is significantly reduce serum uric acid levels, with empagliflozin showing the most pronounced effect (-46.75 μmol/L) (78). In patients with type 2 diabetes mellitus (T2DM), baseline uric acid (UA) levels are closely associated with cardiorenal outcomes and the risk of mortality (79). Elevated serum uric acid (SUA) levels can increase the risk of cardiovascular mortality in patients with chronic kidney disease (CKD) (80) and are associated with increased risks of all-cause mortality and cardiovascular disease (CVD) mortality in diabetic patients (81).

Uric acid can also serve as a key clinical indicator of oxidative stress (82). Oxidative stress increases tissue oxygen consumption and impairs mitochondrial function. Studies have shown that SGLT2is improve mitochondrial biogenesis by activating the AMPK/SIRT1/PGC-1α pathway, thereby reducing oxidative stress (83, 84). Oxidative stress increases tissue oxygen consumption and impairs mitochondrial function. Studies have shown that SGLT2is improve mitochondrial biogenesis by activating the AMPK/SIRT1/PGC-1α pathway, thereby reducing oxidative stress (85, 86). Furthermore, long-term SGLT2i treatment can stimulate renal erythropoietin secretion or hypoxia-inducible factor (HIF), promoting erythropoiesis (87–89). This improvement in renal oxygenation may confer significant renal benefits, as hypoxic injury is a common mechanism leading to adverse renal outcomes (90–92). Several mediation analyses from the EMPA-REG OUTCOME and CANVAS trials have indicated that changes in hematocrit and hemoglobin levels mediate a substantial portion of the benefits of SGLT2is (93, 94), with their effects surpassing those of changes in urate and the urinary albumin-to-creatinine ratio (UACR) in improving renal outcomes (94).

## 3 Clinical significance of metabolic regulation

### 3.1 Cardiovascular protection

SGLT2is have been incorporated into the HF treatment guidelines as a Class I recommended medication and are applicable to all HF patients across the spectrum of ejection fractions (95). Research indicates that SGLT2is can reduce cardiovascular events and mortality in elderly or frail, high-risk T2DM patients with HF (96), decrease HF events in CKD patients (97), and, through combination therapy, improve metabolism in symptomatic adult congenital heart disease (ACHD) patients (98). These studies not only expand the clinical application scope of SGLT2is but also provide in-depth insights into their potential mechanisms of action. SGLT2is can remodel the left ventricle, improving its structure and function. A meta-analysis by Savage et al. demonstrated that SGLT2is can improve left ventricular function in patients with HF, and a trend toward improvement in left atrial-related indices was also observed (99).

Empagliflozin also has similar cardioprotective effects in patients with prediabetes (100). The cardiovascular benefits of SGLT2is are independent of glycemic control, and a 6-month treatment failed to improve left atrial function in high-risk patients (101). Interestingly, one study found that for patients with acute decompensated heart failure (ADHF), empagliflozin treatment for 5 days improved left atrial volume (102). The inconsistency in therapeutic efficacy may be associated with baseline disease conditions and the patient’s own organic changes, which suggests the importance of personalized treatment in the future. Moreover, SGLT2is can reduce NT-proBNP levels in HF patients, with canagliflozin showing the most significant effect (103). In terms of quality of life (QoL), SGLT2is significantly improved quality of life, as assessed by the Kansas City Cardiomyopathy Questionnaire Overall Summary Score (KCCQ-OSS), after 3 months of treatment (104). Compared with HFpEF, empagliflozin is superior in improving exercise capacity and QoL in patients with heart failure with a reduced ejection fraction (HFrEF) (105).

In patients with myocardial infarction (MI), SGLT2is significantly reduced the hospitalization rate for heart failure (HF) (106–109), regardless of whether the MI occurred recently or in the past (110). A meta-analysis by Mukhopadhyay et al. indicated that SGLT2is reduced the risk of major adverse cardiovascular events (MACEs) in patients with T2DM but did not affect MI or stroke (111). A meta-analysis of 13 randomized controlled trials by Liang et al. further confirmed that SGLT2is significantly reduced the risk of nonfatal myocardial infarction by 12% in patients with T2DM but had no significant effect on the risk of nonfatal stroke. Notably, the study by Liang et al. included four studies from the analysis by Mukhopadhyay et al., with a larger sample size, rendering the results more representative. Research by Asham et al. demonstrated that SGLT2is could reduce all-cause mortality (OR, 0.55; 95% CI, 0.38–0.81; P = 0.002; I² = 0%) and improve the left ventricular ejection fraction (SMD, 0.36; 95% CI, 0.02–0.70; P = 0.04; I² = 62%) (108). A recent study by Jia et al. reported for the first time that SGLT2is can reduce the risks of HF combined with cardiovascular death, all-cause mortality, severe arrhythmias, and renal injury while improving left ventricular function (112). This finding contradicts previous findings that SGLT2is do not significantly improve the risks of all-cause mortality, cardiovascular death, or all-cause hospitalization (106, 107, 110). Although there are some differences in the results of various meta-analyses, some studies have been included repeatedly. On the basis of the current evidence, SGLT2is can significantly reduce the HF hospitalization rate in MI patients and may improve all-cause mortality and MACEs, but further precise evaluation is still needed.

SGLT2is also exert cardiovascular protective effects in patients with atrial fibrillation (AF) and arrhythmias. Studies have shown that SGLT2is can reduce the risk of arrhythmias or atrial fibrillation in patients with T2DM (113). Additionally, SGLT2is can decrease all-cause mortality (RR, 0.37; 95% CI, 0.28–0.50), heart failure (RR, 0.66; 95% CI, 0.53–0.83), stroke (RR, 0.76; 95% CI, 0.66–0.88), and cardiovascular mortality (RR, 0.57; 95% CI, 0.44–0.74) in T2DM patients with AF (114). SGLT2i therapy can also prevent the recurrence of AF after catheter ablation in patients with T2DM (115). However, for high-risk patients (such as those with concurrent HF or CKD, although SGLT2is have potential metabolic benefits), SGLT2i therapy does not significantly reduce the risk of AF occurrence (116).

### 3.2 Potential for combination therapy

As an adjunct to insulin therapy, SGLT2i therapy can effectively reduce blood glucose levels, decrease body weight, and reduce insulin dosage requirements in patients with type 1 diabetes (T1D). However, its potential adverse effects, such as hypoglycemia and diabetic ketoacidosis (DKA), require careful evaluation. Multiple clinical studies have shown that there are differences in the safety profiles of various SGLT2is: canagliflozin (100/300 mg) increased the incidence of ketoacidosis-related adverse events during an 18-week treatment period (117); dapagliflozin (5/10 mg) was associated with some mild adverse reactions, such as nasopharyngitis or urinary tract infections, and had a lower incidence of severe hypoglycemia during a 24-week treatment period, although the risk of DKA still warrants caution (118, 119); and the 52-week treatment outcomes of sotagliflozin (400 mg) indicated risks of DKA and hypoglycemia (120, 121). In contrast, the risk of DKA with empagliflozin is dose-dependent (10 mg: 4.3%; 25 mg: 3.3%; 2.5 mg: 0.8%) and does not increase the risk of hypoglycemia (122). Additionally, ipragliflozin (50 mg) did not cause significant safety concerns during the 24-week treatment period (123). Recent studies have shown that the combination of dapagliflozin (10 mg) with a glucagon receptor antagonist (GRA) increases glucose-lowering efficacy and reduces the risk of DKA. However, the study had a small sample size (n=12), and larger-scale trials are needed for validation (124). On the basis of the existing evidence, ipragliflozin demonstrates a superior safety profile, whereas a low dose of empagliflozin (2.5 mg) may be a preferable option for patients at risk of hypoglycemia.

As SGLT2is are glucose-lowering, their combined effects with other hypoglycemic drugs are noteworthy. There may be crosstalk between the mechanisms of action of these hypoglycemic agents, and whether their combination results in additive or diminished effects warrants further exploration to optimize blood glucose management. The combination with GLP-1 receptor agonists does not lead to hypoglycemia, and the effects are independent. Both drugs individually have protective effects on the cardiovascular system and kidneys, and their combination does not interfere with each other (125, 126). However, in the prevention of major adverse cardiovascular and cerebrovascular events (MACCEs) and heart failure, their combination has synergistic effects (127). When combined with pioglitazone, SGLT2is have additive effects in terms of hypoglycemia and weight reduction and further reduce the risk of HF (128). When used in combination with metformin, SGLT2is significantly reduce inflammatory markers through anti-inflammatory mechanisms (129) and do not increase the risk of fractures (130). Moreover, the combination does not affect the cardiovascular benefits of SGLT2is (131). Additionally, triple therapy (SGLT2i + metformin + DPP-4 inhibitor) can provide better glycemic control but may increase the risk of genital infections (132). Therefore, when a combination regimen is selected clinically, it is necessary to balance efficacy and safety and to individualize treatment strategies.

The combination of SGLT2is with renin–angiotensin system blockers, including ACE inhibitors and ARBs, has synergistic effects on the treatment of type 2 diabetes mellitus (T2DM). This combination not only enhances glucose-lowering and blood pressure-lowering effects but also significantly improves renal outcomes, although it may increase the risk of hypoglycemia and genital infections (133, 134). In patients with diabetic kidney disease (DKD) and CKD, the combination of SGLT2is with ACEIs/ARBs effectively protects renal function and slows the progression of kidney disease (135, 136). Furthermore, the combination of SGLT2is with mineralocorticoid receptor antagonists (MRAs) provides additional cardiovascular benefits for patients with T2DM and CKD while reducing the risk of hyperkalemia (137). More notably, combination therapy with SGLT2i and angiotensin receptor–neprilysin inhibitor (ARNI) has demonstrated significant efficacy in patients with HFrEF: it reduces the risk of the composite endpoint of heart failure hospitalization or cardiovascular death by 32%, decreases cardiovascular death by 36%, and decreases all-cause mortality by 28%, although it may increase the risk of hypovolemia (138). Combination with sacubitril-valsartan (SV) can also enhance cardiovascular protection in HFrEF patients (139). These findings provide important evidence for optimizing cardiorenal protection strategies in patients with T2DM (**Table 1**).

**Table 1:** SGLT2i combination therapy.

References	Type of study	Disease	Drugs	Combination medication	Effect
(117–123)	RCT	T1D	Kagliflozin, Dapagliflozin, Soragliflozin, Empagliflozin, Ipagliflozin	insulin	Reduce fasting blood sugar HbA1c, Weight, pancreatic islet basal/recommended dose, with risks of hypoglycemia and DKA.
(124)	RCT	T1D	Dapagliflozin+GRA (Valacizumab)	Insulin	GRA enhances the therapeutic effect of SGLT2i on T1D while reducing the risk of DKA
(125–127)	meta-analysis	T2D	SGLT2is	GLP-1	Significantly reduce blood sugar and systolic blood pressure without increasing the risk of hypoglycemia. However, the effect of the combined application is not additive, and single-use has a better effect. Combined use is beneficial for the prevention of MACCE and HF.
(128)	meta-analysis	T2D	SGLT2is	Pioglitazone	The effect of reducing HbA1c, body weight, and SBP is better than using SGLT2i alone, reducing the risk of heart failure.
(129–131)	meta-analysis	T2D	SGLT2is	Metformin	Lowering blood sugar and lowering levels of inflammatory factors such as CRP, TNF - α, UA, leptin, etc., does not lead to fracture risk and does not affect the CV benefits of SGLT2 inhibitors.
(132)	meta-analysis	T2D	SGLT2is	Metformin+DPP-4 inhibitor	Significantly improves blood sugar, weight, and blood pressure, with better efficacy than the combination therapy of SGLT2 inhibitors and metformin, but increases the risk of reproductive tract infections.
(133, 134)	meta-analysis	T2D	SGLT2is	ACEI/ARB	Better blood sugar and blood pressure control, improved kidney outcomes, alleviated long-term kidney function, increased risk of hypoglycemia and genital infections
(135)	meta-analysis	DKD	SGLT2is	ACEI/ARB	Significantly reduced albuminuria, HbA1c, and SBP, delayed progression to end-stage renal disease (ESRD), and had no significant impact on the incidence of kidney-related adverse events or kidney-related mortality
(136)	meta-analysis	CKD	SGLT2is	ACEI/ARB	Significantly reduced 24-hour urinary albumin excretion rate (24-hour UAE) and creatinine elevation rate, delayed progression to end-stage renal disease, with no significant impact on the incidence of renal-related adverse events or renal-related mortality
(137)	meta-analysis	T2D+CKD	SGLT2is	MRA	Reduce cardiovascular (CV) events and lower the risk of hyperkalemia.
(138)	meta-analysis	HFrEF	SGLT2is	ARNI	Cardiovascular mortality decreased by 36% and all-cause mortality decreased by 28%. Although the estimated treatment effect is a 55% increase in blood volume deficiency, more attention should be paid to blood volume deficiency.
(139)	meta-analysis	HFrEF	SGLT2is	Sacubitril Valsartan (SV)	Enhance the cardiovascular protective effect of SGLT2i.

## 4 Future directions and challenges

Although SGLT2is have demonstrated significant pleiotropic effects in the treatment of T2DM and related complications, their clinical application still faces numerous challenges. Future research needs to further optimize treatment strategies and explore the underlying mechanisms involved. Currently, the efficacy of SGLT2is is heterogeneous across different patient populations, and future studies should focus on screening biomarkers to predict patients’ responses to SGLT2is. Additionally, precision medicine models based on artificial intelligence and big data may help optimize dosing regimens and achieve individualized treatment. The pleiotropic mechanisms of SGLT2is have not been fully elucidated, particularly the specific pathways through which they affect adipose tissue remodeling, myocardial energy metabolism, and renal protection. Future research should integrate metabolomics, imaging, and molecular biology techniques to further elucidate the underlying mechanisms of SGLT2is in organ protection. Additionally, the impact of SGLT2is on emerging fields, such as the gut microbiota and immune regulation, also warrants exploration.

In conclusion, the potential of SGLT2is in the management of T2DM remains to be fully explored. Future research should integrate basic and clinical sciences to advance the development of personalized treatment strategies and address existing challenges, thereby maximizing long-term benefits for patients.

## 5 Summary

SGLT2is exhibit extensive pleiotropic effects in the treatment of T2DM and its complications through a unique mechanism. This article systematically reviews the significant roles of SGLT2is in metabolic regulation, cardiovascular protection, and combination therapy. It also proposes that, on the basis of the metabolic pleiotropy of SGLT2is, the development of individualized treatment strategies is the future trend in the application of SGLT2is. The effects of combination therapy can be synergistic, additive, or independent and carry risks of adverse events such as hypoglycemia, DKA, and local tissue inflammation. Therefore, evaluating the treatment duration, as well as the dosage form and dosage of the medication, are critical factors in targeted therapy. Future research should further explore the precise application of SGLT2is in different populations as well as their long-term safety and their protective mechanisms for specific organs.

In summary, SGLT2is are not only efficient hypoglycemic agents but also multifunctional metabolic modulators, offering a new therapeutic paradigm for the management of T2DM and its complications. With further research, the clinical application prospects of SGLT2is will become even broader, resulting in increased benefits for patients.
